# Extracellular vesicle integrins act as a nexus for platelet adhesion in cerebral microvessels

**DOI:** 10.1038/s41598-019-52127-3

**Published:** 2019-11-01

**Authors:** Zsolt Bagi, Yvonne Couch, Zuzana Broskova, Francisco Perez-Balderas, Tianrong Yeo, Simon Davis, Roman Fischer, Nicola R. Sibson, Benjamin G. Davis, Daniel C. Anthony

**Affiliations:** 1 Department of Pharmacology, University of Oxford, Oxford, OX1 3QT United Kingdom; 2 RDM-Investigative Medicine, University of Oxford, Oxford, OX3 7LJ United Kingdom; 3 Chemistry Research Laboratory, University of Oxford, Oxford, OX1 3TA United Kingdom; 4 Gray Institute for Radiation Oncology and Biology, University of Oxford, Oxford, OX3 7DQ United Kingdom; 5 Department of Physiology, Medical College of Georgia, Augusta University, Augusta, GA 30912 USA; 6 TDI Mass Spectrometry Laboratory, Target Discovery Institute, Nuffield Department of Medicine, University of Oxford, Roosevelt Drive, Oxford, OX3 7FZ UK

**Keywords:** Platelets, Molecular medicine, Inflammation

## Abstract

Circulating extracellular vesicles (EVs) regulate signaling pathways via receptor-ligand interactions and content delivery, after attachment or internalization by endothelial cells. However, they originate from diverse cell populations and are heterogeneous in composition. To determine the effects of specific surface molecules, the use of synthetic EV mimetics permits the study of specific EV receptor-ligand interactions. Here, we used endogenous EVs derived from the circulation of rats, as well as ligand-decorated synthetic microparticles (MPs) to examine the role of integrin αvβ3 in platelet adhesion under flow in structurally intact cerebral arteries. At an intraluminal pressure of 50 mmHg and flow rate of 10 µl/min, platelets were delivered to the artery lumen and imaged with whole-field fluorescent microscopy. Under basal conditions very few platelets bound to the endothelium. However, adhesion events were markedly increased following the introduction of arginine-glycine-aspartate (RGD)-labelled synthetic MPs or endogenously-derived EVs from experimental stroke animals carrying excess RGD proteins, including vitronectin, CD40-ligand and thrombospondin-1. These data, which were generated in a dynamic and physiologically relevant system, demonstrate the importance of vesicle-carried RGD ligands in platelet adherence to the cerebrovascular endothelium and highlight the ability of synthetic EVs to isolate and identify key components of the molecular handshake between EVs and their targets.

## Introduction

Platelet interactions with the microvascular endothelium have emerged as an important pathological process during cerebral inflammation^1,2^. We have previously demonstrated platelet binding to the arterial microvascular endothelium *in vivo*, in a mouse model of cerebral malaria^3^. The nature of the molecular mechanisms that govern platelet adhesion to the intact arterial endothelium remain unknown, but it is understood that an increase in circulating extracellular vesicles (EVs), including platelet-derived extracellular vesicles, can contribute to a hypercoaggulative state^4^. However, the EV signature responsible for this state is yet to be discovered.

Arginine-glycine-aspartate (RGD)-motif recognizing integrins, such as αvβ3, are constitutively expressed by microvascular endothelial cells and play a key role in physiological and pathological angiogenesis^5^. As part of this process, αvβ3 mediates the interaction between endothelial cells and subendothelial extracellular matrix proteins through binding to exposed RGD sequences. However, αvβ3 is also expressed on the luminal surface of the endothelium^6,7^. Early *in vitro* studies by Gawaz *et al*.^6,8^ showed that cultured cells co-transfected with human αIIb and β3 integrins co-aggregate with endothelial cells bearing integrin αvβ3. This suggests that the expression of αvβ3 is key for the binding of cells to the activated endothelium. Activating the endothelium using plasma from patients with myocardial infarction has been shown to result in increased adherence of platelets^8^. Under these conditions, the adherence of platelets to the endothelial monolayer was blocked by antibodies against integrin αvβ3 or by RGD peptides, which highlights the involvement of both endothelial integrin αvβ3 and exposed RGD peptides. In their study, Gawaz and colleagues also noted that platelet-derived EVs can also bind to endothelial cells, but it was not clear whether EV-endothelial cell binding might alter platelet binding to endothelial cells^8^. Studies have subsequently shown that circulating EVs derived from platelets are capable of promoting platelet adhesion^9^, but the molecular basis for the platelet-endothelial interaction and the role of EVs in this process has remained largely unexplored.

In this study, we set out to develop an optical imaging approach to investigate the adhesive properties of integrin αvβ3 on the surface of the intact endothelium of small cerebral arteries under flow conditions, at relevant physiological pressures. We sought to use this technique to assess the role of vesicle-RGD integrin-binding motifs in platelet-endothelium interactions, specifically platelet adhesion. As well as using EVs from whole blood, we also used multivalent synthetic EVs which enabled us to investigate the more precise interactions between EVs and target in a manner that, hitherto, has not been possible with intact EVs and neutralizing agents. The data obtained reveals that this *ex vivo* approach provides a method to identify a wide-range of EV-vessel interactions at a molecular level, with accurate temporal and spatial resolution. In this way it is now possible to discover how individual ligand-receptor interactions contribute to EV-mediated adhesion events.

## Materials and Methods

### Synthesis of RGD peptides and labelling of microparticles

H-GCRGDC-NH_2_ (cRGD) and H-GCRGGC-NH_2_ (with scrambled peptide sequence, scrRGD) were synthesized by solid phase peptide synthesis and cyclization was performed in resin by iodine oxidation. We synthetized carboxy functionalized microparticles (MP) labelled with Alexa-Fluor-488 (~0.7–1.0 µm)^10^ or used tosylactivated Dynabeads (2.8 µm, Invitrogen) to covalently bind cRGD or scrRGD peptides as well as the recombinant mouse vitronectin (VTN) protein (AbCam) on their surface. Briefly, carboxy functionalized MPs (2 mg) were pre-activated by EDC/sulfoNHS and mixed with 0.1 mg of cRGD or scrRGD peptide for 24 hours at room temperature (in NaHCO_3_ buffer pH 8.3) to size cRGD-MPs or scrRGD-MPs. MPs were collected and washed in phosphate buffer saline (PBS) containing 0.1% Tween-20 by using a Dynal magnet (Invitrogen). Tosylactivated Dynabeads (2 mg) were reacted with 0.1 mg of vitronectin following the protocol supplied by the manufacturer, to size VTN-MPs. All samples were re-suspended in physiological salt solution (PSS) on the day of the experiments. A Qifikit (Dako) assay kit was used to quantify peptide/protein labelling, as per the manufacturer’s instructions (*see* Supplementary Fig. 1). Briefly, Qifikit beads (10 µm) with well-defined quantities of mouse monoclonal antibodies were subsequently labeled with Alexa Fluor 647 goat-anti-mouse IgG antibody. In parallel, cRGD-MPs or scrRGD-MPs were incubated with TCEP.HCl and Alexa-Fluor-647 C_2_ maleimide for 30 minutes. MPs were pelleted using a Dynal magnet, and washed three times in PBS. VTN-MPs were incubated with rat-anti-mouse vitronectin IgG antibody (R&D systems, clone 347317) for 2 hours. MPs were collected with a magnet, re-suspended in PSS and incubated with Alexa-Fluor-647 goat-anti-rat antibody (Invitrogen). Flow cytometry analysis was performed with BD LSRII cytometer (BD Biosciences). Supplementary Fig. 1 shows the effective labeling of synthetic MPs (Supplemental Fig. 1). It should be noted that using this assay the number of surface molecules can be underestimated owing to cross-linking of adjacent primary antibodies.

**Figure 1:**
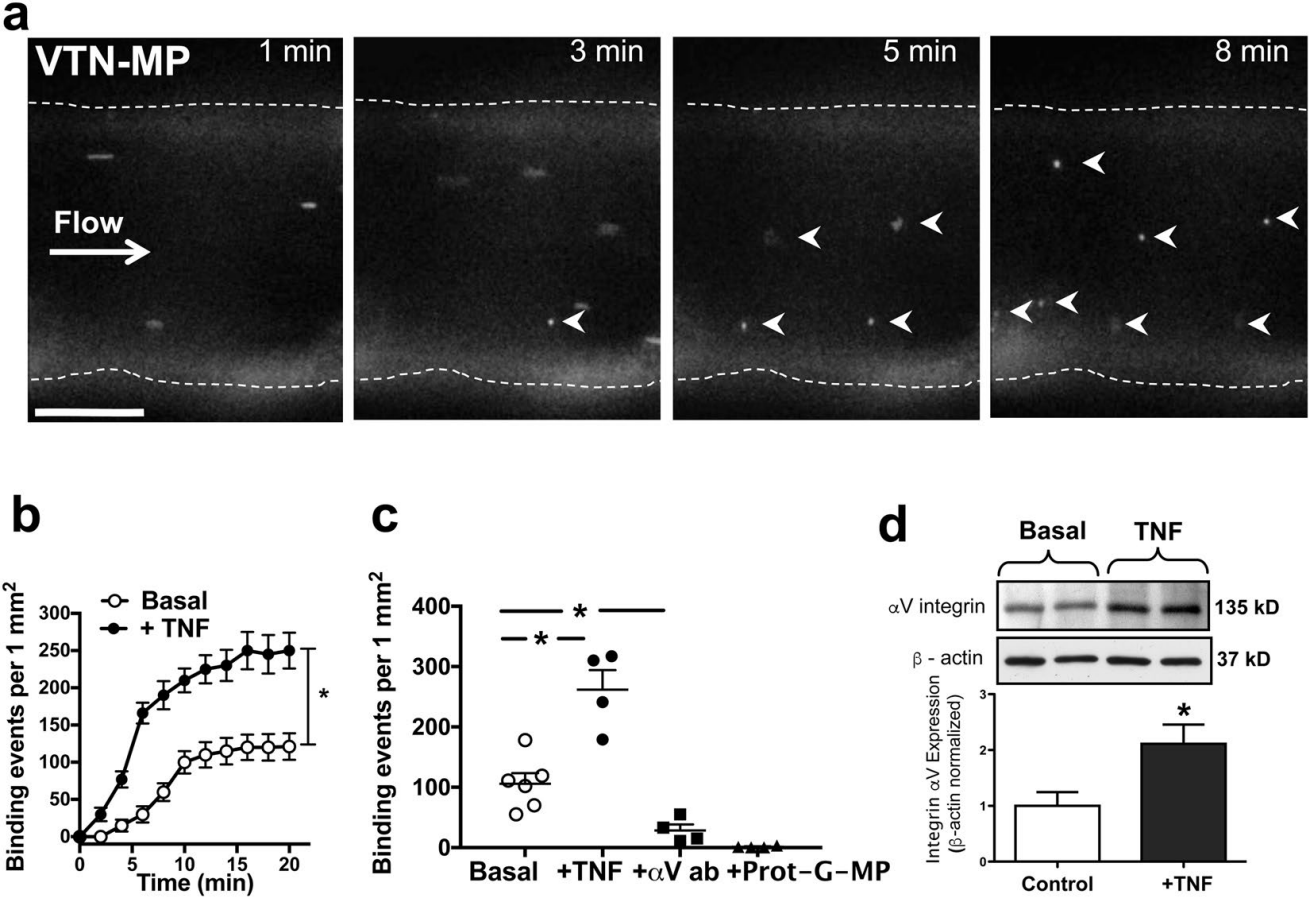
Microparticle (MP) binding under flow in the isolated middle cerebral artery. (**a**) Fluorescence images of representative time-lapse recording demonstrate firm binding of VTN-MPs (0.1 µg/ml) on the luminal surface of the isolated mouse MCA in the presence of physiological pressure and flow (white arrowheads show stationary MPs). Scale bars: 50 μm. (**b**) Summary data (n = 4–6) show the rate and number of binding events of VTN-MPs in the absence (basal) or presence of TNF (7 ng/ml, 4-hour), or after incubating with αV integrin blocking antibody, or after delivery of Protein G conjugated MPs (**c,d**) Representative images and summary data of Western immunoblot shows increased expression of αV integrin on TNF-exposed mouse MCA. Data are shown as mean ± S.E.M; *p < 0.05.

### Imaging of MP binding *ex vivo* in the middle cerebral artery under flow

Experiments were carried out in 8–12 week-old, male C56BL/6 mice and 12–14 week-old male, Wistar rats. All animal experimental procedures performed in this study were in compliance with the European Convention for the Protection of Vertebrate Animals used for Experimental and other Scientific Purposes. The work involving experimental animals was conducted under UK Home Office licence 30/3076. The animals were housed in the animal care facility and accessed rodent chow and tap water *ad libitum* with a 12-h light:dark cycle. Mice were euthanized with CO_2_ and the brain was immediately removed and placed in ice-cold Krebs solution (in mmol/l): 110.0 NaCl, 5.0 KCl, 1.25 CaCl_2_, 1.0 MgSO_4_, 1.0 KH_2_PO_4_, 5.5 D-glucose, and 24.0 NaHCO_3_), equilibrated with a gas mixture of 10% O_2_–5% CO_2_-balanced nitrogen, at pH 7.4. With the use of microsurgical instruments and an operating microscope the middle cerebral artery (MCA, ~1.0 mm in length) was isolated and transferred into an organ chamber containing two glass micropipettes filled with Krebs solution. The vessel was cannulated at both ends and the micropipettes were connected with silicone tubing to a hydrostatic reservoir to set the intraluminal pressure to 50 mmHg. Intraluminal flow was established by adjusting the inflow and outflow pressures in opposite directions, but to equal flow rates until a constant 10 µl/min flow was established through the vessel. Flow was measured with a ball-type flow meter (Omega). MPs (1 µg/ml, prepared freshly from 1 mg/ml stock solution, 1:1000 dilution) were administered intraluminally through the cannulating glass capillary at constant flow rate (10 µl/min). The MPs were visualized with whole-field fluorescence measurements by using an ultra-fast filter switching-based imaging system (Oligochrome, TILL Photonics) on an upright microscope (Nikon Eclipse FN1), using a water-dipping objective (40×, NA: 0.8) and illuminated with 475/35 nm light. A series of high-resolution images were acquired with a cooled EM CCD camera (LucaS, Andor) at 15 Hz. Time-lapse images were analyzed offline (Arivis Browser, TILL Photonics). The endothelial surface bound MPs were manually counted and presented as binding events per luminal surface area of the isolated MCA. Presence of divalent cations, such as Mn^2+^ and Mg^2+^ are required for firm binding to integrin αvβ3, but studies also have shown that these divalent cations do not seem to change the structure and activation state of αvβ3 unless a high-affinity RGD ligand is present^11^. Our binding experiments were performed in physiological salt solution containing Mg^2+^, which alone was shown to support integrin-mediated interactions^12^.

In separate experiments, the MCA was incubated with or without TNF (7 ng/ml, for 4 hours) and MP bindings were reassessed in the absence and presence of an adhesion blocking anti-αv antibody (Clone RMV-7, BD Pharmingen, 1 µg/ml). In this protocol, for controls we used MPs conjugated with integrin αv indifferent protein, specifically protein G-MPs (2.8 µm, Dynabeads ^TM^ Protein G, Invitrogen). We also tested whether the vascular endothelium remains functionally intact after delivering MPs. To this end, vasodilation in response to the endothelium-dependent agonist, acetylcholine was measured, before and after delivering the MP. Dilations greater than 50% indicated functionally intact endothelium (Supplementary Fig. 2). In addition, to further demonstrate that binding of MPs occurs to the structurally intact endothelial cell surface we used conventional immune-histology techniques. In brief, after the imaging experiments, MCA was fixed with 4% paraformaldehyde and embedded in gelatin. Sections of 50-µm thickness were cut with a vibratome and immuno-labeled with an anti-CD31 antibody (AbCam). A Cy5 secondary antibody (Jackson Immunoresearch) was used for detection. Florescent images were collected with a structured illumination fluorescent microscope (Zeiss AxioImager M2, Apotome2).

### Western blotting

SDS electrophoresis and subsequent Western immunoblotting was carried out in isolated MCA. MCA was incubated with TNF (7 ng/ml, for 4 hours) or vehicle and then was snap-frozen in liquid nitrogen. MCA was homogenized in 20 µl ice-cold RIPA buffer (Sigma) and additional 20 µl of Laemmli buffer were added (Sigma). Anti-αv integrin (1:1000, ab112487, AbCam) and anti-β-actin primary (1:5000, clone AC-15, Sigma, for loading control and normalizing), and corresponding horseradish peroxidase-labeled secondary antibodies were used for detection. Chemiluminescence was visualized and quantified with ChemiDoc™ MP system (BioRad).

### Imaging of platelet adhesion in the *ex vivo* MCA under flow

Washed platelets were freshly isolated from the whole blood of rats, treated with 200 µM PGE_2_ and 1 unit/ml apyrase to prevent activation, as described previously^13^. The washed platelets were directly labeled with carboxyfluorescein succinimidyl ester (CFSE), further diluted in Tyrode’s solution (to reach final concentration of 1.5 × 10^6^/ml) and were delivered intraluminally into the pressurized (70 mmHg) MCA, at constant flow rate (10 µl/min). Individual binding events of platelets were recorded with high-speed fluorescent microscopy as described above. Platelet binding events were also recorded after the prior administration and incubation (10 min) with cRGD-MP, scrRGD-MP or cRGD peptide solution (100 μg/ml). In separate experiments the isolated MCA was incubated with TNF (7 ng/ml for 4 hours) and platelet adhesion was assessed. Platelet adhesion was also observed in endothelium-denuded arteries. Endothelium removal was achieved by perfusion the blood vessels with air and confirmed by the lack of acetylcholine-induced vasodilation.

### Isolation of EVs from the serum of rats after ischemic stroke

A transient middle cerebral artery occlusion (tMCAO) was performed in 12- week-old male Wistar rats. Briefly, under isoflurane anesthesia, an occluding filament was introduced into the internal carotid artery and advanced to occlude the MCA completely for 90 minutes. For controls, sham-operated animals were used without inserting the occluding filament. After 48 hours the rats were euthanized with CO_2_ and the brain was placed in ice-cold Kreb’s soultion. Ischemic brain damage was confirmed by triphenyltetrazolium chloride (TTC) staining. Extracellular vesicles (EVs) were isolated from the serum of rats using ultracentrifugation as described earlier^14^. Size distribution and concentration of EVs were analyzed by laser scattering video-microscopy (ZetaView, Microtrac). Western blot analysis for protein content of EVs was also performed. Briefly, EVs were lysed in RIPA buffer and equal amounts of protein were loaded for SDS electrophoresis. After blotting, anti-thrombospondin-1 (1:1000, [A6.1], AbCam), anti-vitronectin (1:200, H-270, Santa Cruz Biotechnology) and anti-CD40 ligand antibodies (1:500, ab2391, AbCam), and corresponding horseradish peroxidase labeled secondary antibodies were used for detection. Signals were revealed with chemiluminescence and visualized autoradiographically. EVs were re-suspended in Tyrode’s solution and were delivered into the *ex vivo* pressurized cerebral artery. As above, washed and CFSE labeled platelets were subsequently administered intraluminally and binding events were recorded.

### Liquid chromatography mass spectrometry

EVs were harvested from the serum of patients with acute ischemic stroke (<24 hours of the clinical diagnosis) and age-matched controls (OXVASC Study, University of Oxford)^15^. In addition EVs were harvested from the serum of tMCAO animals, as described above. Liquid chromatography – mass spectrometry/mass spectrometry (LC-MS/MS) analysis was performed in technical duplicates using a Dionex Ultimate 3000 UPLC coupled on-line to a Q Exactive HF mass spectrometer (Thermo Scientific). Samples were separated on an EASY-Spray PepMap C18 column (500 mm × 75 µm, 2 µm particle size, Thermo Scientific) over a 60 minute gradient of 2–35% acetonitrile in 5% DMSO 0.1% formic acid at 250 nl/min. MS1 scans were acquired at a resolution of 60,000 at 200 m/z and the top 15 most abundant precursor ions were selected for HCD fragmentation. Protein quantitation was performed using Progenesis QI for Proteomics (Non-linear Dynamics, version 2.0). MS/MS data was searched using Mascot (Matrix Science, version 2.5.1) against the human Swissprot database (retrieval date 15.10.14) allowing 1 missed cleavage. Mass tolerances were 10 ppm for precursor and 0.05 Da for fragment masses. Carbamidomethylation of cysteine was set as a fixed modification. Oxidation of methionine, deamidation of asparagine and glutamine were set as variable modifications. Identified peptides scoring below 20 following application of a 1% false discovery rate were discarded and the Mascot search results were imported back into Progenesis for label-free protein quantitation.

### Statistical analysis

Statistical analyses were performed using GraphPad Prism software. One-way Analysis of Variance ANOVA with Tukey post-hoc test (comparison of multiple groups) or Student’s T-test (comparison of two groups) was performed. p < 0.05 were considered statistically significant. Comparaision

## Results

### Synthetic microparticles (MPs) decorated with vitronectin (VTN) bind to an activated endothelium in an integrin-dependent manner

**Figure 2:**
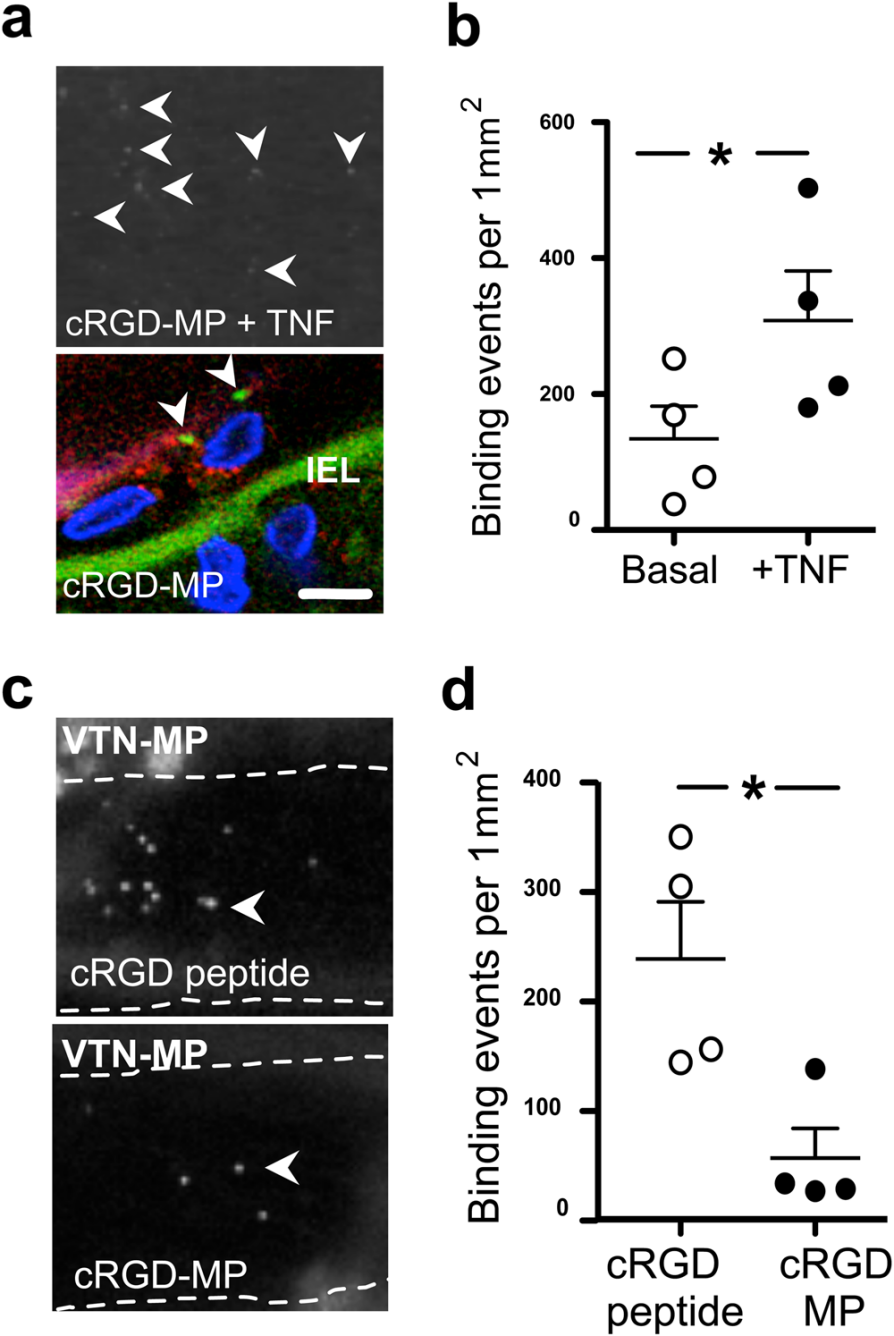
Modulation of RGD-endothelial interactions by microparticle-carried ligands. (**a**) Representative images show localization of cRGD-MPs (white arrowheads - upper panel; shown in green with arrowheads - lower panel) and CD-31 staining (red - lower panel) on TNF-activated endothelial cells (blue - DAPI). (**b**) Bindings of cRGD-MPs (0.1 µg/ml) in isolated, pressurized MCA, before and after incubation with TNF. (**c**) Representative images show VTN-MP bindings in TNF-exposed MCA with prior intraluminal delivery of cRGD peptide (100 μg/ml) or cRGD-MPs (0.1 µg/ml). (**d**) Summary data of VTN-MP bindings in TNF-exposed MCA with cRGD peptide or cRGD-MPs. IEL: Internal Elastic Lamina. Scale bar: 10 μm. Data are mean ± SEM; *p < 0.05.

Mouse middle cerebral arteries (MCA) were isolated, cannulated with a glass capillary and pressurized. Under a physiologically relevant constant intraluminal pressure (50 mmHg) and flow rate (10 µl/min), MPs were delivered and imaged within the vascular lumen using high-speed, whole-field fluorescent microscopy. This technique allowed us real-time detection of individual binding events of the synthetic MPs to the luminal surface of endothelial cells (Fig. 1a). Using this approach, we detected increased binding events of VTN-MPs over the 20-minute observation period (Fig. 1a,b). VTN was chosen as it contains an RGD sequence and we suspect that EVs expressing VTN contribute to post-stroke pathology. Consistent with anticipated inflammatory cytokine responses^8^, we found that exposure of isolated mouse MCA to the pro-inflammatory cytokine, tumor necrosis factor α (TNF, 7 ng/ml, for 4 hours) that is expressed in stroke lesions, increased the protein expression of integrin αv (Fig. 1d). Correspondingly, the number of endothelial surface binding events of VTN-MP (Fig. 1c) and also cRGD-MP (Fig. 2a,b) increased significantly in TNF-exposed vessels, when compared to the binding of an integrin αv-indifferent protein, protein-G coated MPs. To assess the binding specificity of VTN-MP we used an anti-αv antibody (Clone RMV-7, BD Pharmingen) developed against the RGD-recognizing extracellular domain of integrin αv. The RMV-7 antibody has been shown to strongly inhibit binding of VTN and fibronectin to integrin αv-coated surfaces^16^. We found that administration of the anti-αv antibody significantly reduced binding of VTN-MPs to the TNF-activated endothelium (Fig. 1c).

### Multivalent interaction mediates RGD microparticle binding to the vascular endothelium

Given the ability of the RMV-7 antibody to suppress VTN-MPs binding, we next explored the role of the RGD sequence in promoting MP clustering and binding. To achieve this, binding of VTN-MPs was assessed in the presence of cRGD peptide solution alone (a ‘monovalent’ competing probe) or cRGD-MPs (as a ‘multivalent’ competing probe). We found that prior administration and presence of cRGD peptide solution (100 μg/ml, an efficient inhibitory concentration used previously^17^) had no significant effect on increased VTN-MP binding in TNF-exposed arteries. However, administration of multivalent cRGD-MP, which also binds both resting and TNF-activated endothelium (Fig. 2a,b), markedly reduced VTN-MP binding (Fig. 2c,d).

### MPs carrying RGD motifs promote platelet adherence to the vascular endothelium

**Figure 3:**
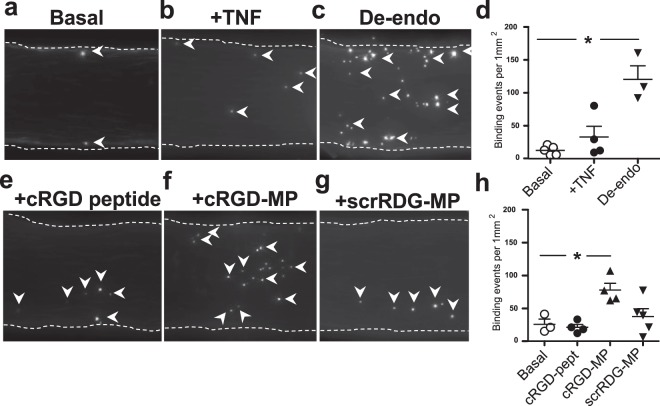
Platelet adhesion is facilitated by synthetic RGD-MPs in the cerebral artery. Fluorescence images of representative time-lapse recording demonstrate firm binding of CFSE-labelled platelets on the luminal surface of the isolated mouse MCA in the presence of physiological pressure and flow (white arrowheads show stationary platelets). (**a–d**) Platelet adhesion was measured under flow in the absence (basal) or presence of TNF (7 ng/ml, 4-hour), and after the endothelium was removed with air (De-endo), as well as (**e–h**) in the presence and prior intraluminal loading with cRGD peptide (100 μg/ml), cRGD-MPs or scrRDG-MPs (0.1 µg/ml, each). Data are mean ± SEM; *p < 0.05.

After establishing the involvement of the RGD sequence in binding *per se*, we then investigated and manipulated adhesion of platelets in the *ex vivo* pressurized MCA system under physiologically relevant flow conditions. We observed only a small number of platelets adhering to un-stimulated vessel lumen under flow (Fig. 3a–d). TNF increased the expression of integrin αv in the MCA, but only marginally increased platelet adhesion under flow (Fig. 3b–d). The number of adhered platelets only increased after the physical removal of endothelium (Fig. 3c,d). Thus, we concluded that resting platelets do not firmly adhere to the TNF-activated endothelium under flow conditions. We therefore explored the possibility that other mechanisms are necessary to mediate firm platelet adhesion in the inflammation-activated cerebral artery endothelium^3^. As platelet integrins, such as αIIbβ3 recognize the RGD peptide sequence, we assessed whether RGD-mediated polyvalent interactions are able to promote platelet adhesion, with MPs acting as a nexus for platelet-endothelial cell interactions. While the presence of cRGD peptide solution did not enhance or prevent platelet adhesion (Fig. 3e–h), we found that administration and presence of cRGD-MPs elicited a three-fold increase in platelet adherence to the surface of the MCA when compared to cRGD peptide alone (Fig. 3f–h). Administration of the scrambled peptide-carrying (scrRGD)-MPs had no significant effect (Fig. 3g,h). Thus, while platelets do not seem to firmly adhere to the inflammation-activated endothelium, multivalent EV-mimetic MPs carrying RGD motifs promote efficient platelet adherence. In these protocols, we used larger synthetic MPs as mimetics of EVs, whose size ranges from 0.05 to 1 µm. Employing these MPs was a methodological compromise to be able to efficiently conjugate ligands and visualize them in the lumen of blood vessels. It is likely that the larger size of these synthetic EVs will increase the number of surface RGD ligands, potentially not representing endogenous EVs. However, the aim of this study was to determine, mechanistically, whether there was the potential for multivalent RGD-mediated binding of platelets to the intact endothelium.

### Extracellular vesicles (EVs) carrying RGD proteins promote platelet adherence to the intact endothelium after ischemic stroke

**Figure 4:**
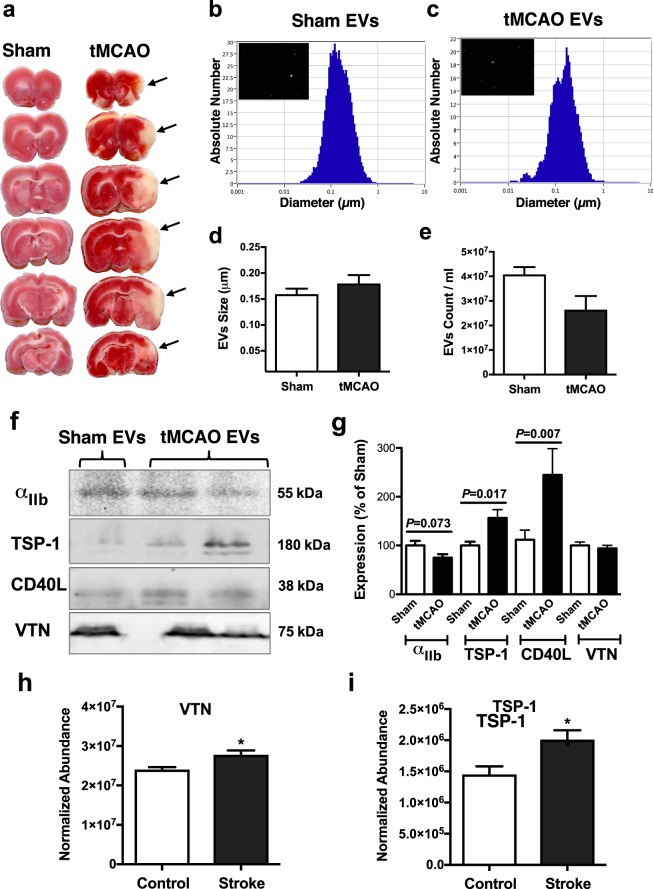
*RGD protein expression in extracellular vesicles (EVs) in ischemic stroke*. (**a**) Triphenyltetrazolium chloride-stained brain slices of rats after tMCAO show ischemic brain areas (outlined – right panel). EVs were isolated and measured using light scattering technology in the serum of sham (**b**) and tMCAO (**c**) animals. Quantification was performed for both size (**d**) and number of EVs/ml (**e**). Representative Western immunoblots (**f**) and summary of densitometry data (**g**) show changes in expression of integrin αIIb, thrombospondin-1 (TSP-1), CD40 ligand (CD40L) and vitronectin (VTN) in EVs isolated from sham-operated or tMCAO rats. Average-fold change in peptide sequences identified as vitronectin (**h**) and thrombospondin-1 (**i**) in EVs harvested from patients with ischemic stroke and age-matched controls, as measured using LC-MS/MS. Data are mean ± SEM; *p < 0.05.

**Figure 5:**
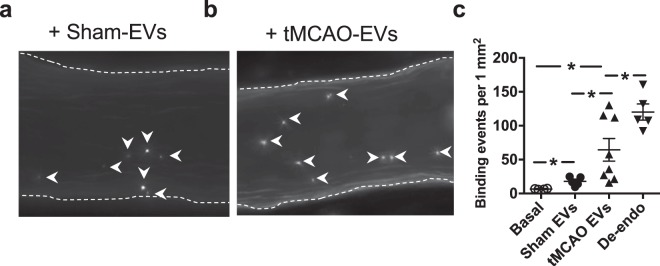
*RGD protein bearing extracellular vesicles (EVs) promote platelet adhesion under flow*. (**a,b**) Fluorescence images of representative time-lapse recording demonstrate firm binding of CFSE-labelled platelets on the luminal surface of the isolated mouse MCA pre-treated with EVs isolated from sham and tMCAO animals, and in the presence of physiological pressure and flow (white arrowheads show stationary platelets). (**c**) Summary data show adhered platelets (white arrow heads) under basal condition, and after the endothelium was removed with air (De-endo), or when sham EVs or EVs from tMCAO were delivered prior to platelets. Data are mean ± SEM, *p < 0.05.

Based on our present findings, we hypothesized that platelet adhesion to the arterial microvascular endothelium may be mediated through RGD-bearing proteins. Platelet adhesion is known to be a mechanism by which platelets mediate vascular inflammation, a key event in the post-ischemic brain. To provide further pathophysiological relevance to our observations, we isolated EVs from the serum of rats with cerebral ischemia-reperfusion injury, induced by transient occlusion of the MCA (tMCAO) (Fig. 4a). Whilst the concentration and size distribution of EVs were similar in the two groups (Figs. 4b–e) EVs from tMCAO rat serum exhibited an increased expression of proteins with RGD sequences, such as thrombospondin-1 and CD40 ligand (Fig. 4f–g). We also confirmed that thrombospondin-1 and vitronectin are expressed on circulating endogenous EVs in human ischemic stroke (Fig. 4h,i). Correspondingly, when EVs from tMCAO rats were delivered into the lumen of the MCA of a normal rat, we found that the subsequent platelet adhesion to the endothelial surface was significantly increased (Fig. 5a–c) compared to that of EVs from sham operated rats. Proteomic analysis of the levels of expression in tMCAO and sham EV populations revealed a partial separation by principal component analysis (PCA) and a separation by orthogonal partial-least square determinant analysis  (OPLS-DA) of with a sensitivity and specificity of 0.79 using the 220 proteins that were detectable and conserved in both populations (Supplementary Fig. 3).

## Discussion

In this study we employed an optical imaging approach to explore the interactions between EVs, platelets and the vascular endothelium. We were able to demonstrate, for the first time, that EVs are able to act as a nexus for platelet-endothelial interactions. Using synthetic EVs (MPs), we established that this occurs via RGD-integrin interactions, in the absence of any other ligands. By using intact vessels under physiologically relevant conditions, we were able to demonstrate that this occurs under flow, not just under the static conditions used in standard cell culture. The use of soluble, univalent cRGD did not alter platelet binding, effectively highlighting the multivalent utility of EV-mimetics to better understand the function of cell-derived EVs in normal physiology and pathophysiology. Thus, we propose that RGD-mediated EV-bridging between platelets and the activated endothelium represents a key potential mechanism by which platelet adhesion to the intact endothelium occurs during pathology.

In previous studies, αvβ3-targeted fluorescent quantum dots have been used to directly visualize and assess the role of endothelial αvβ3 during tumor angiogenesis using RGD peptide-coated nanoprobes^18,19^. However, it has not thus far been possible to selectively detect luminally-expressed αvβ3 owing to the small size of the probes and their tendency to be actively and non-specifically taken up by endothelial cells. As such, functional studies of integrin-mediated binding have been limited. As an alternative technique, microbubbles were covalently bound to the RGD peptide and visualized with ultrasound in a murine model of mammary carcinoma^20^. However, the maximal achievable spatial resolution limits the clinical utility of this technique, especially when compared to molecular imaging in the small vessel lumen^21^. Therefore, in order to selectively target and assess the adhesive properties of endothelial αvβ3, an imaging approach with high resolution, but without ligand uptake, is essential.

In our own previous studies we have employed paramagnetic microparticles (MPs), conjugated to antibodies or peptides, to selectively target endothelial cell adhesion molecules such as E-selectin and vascular cell adhesion molecule-1^22^. These were successfully used to probe *in vivo* experimental cerebral inflammation^23^ and to image adhesion of activated platelets^3^. This highlighted the utility of these agents for the detection of low density and difficult-to-access functional epitopes *in vivo* and *ex vivo*. Results from the present study demonstrate the unique suitability of RGD protein/peptide-decorated MPs to target and quantify αvβ3 integrins that are expressed on the luminal aspect of the cerebrovascular endothelium, under physiologically relevant conditions *ex vivo*. We found that VTN and cRGD-peptide carrying MPs firmly bind on the luminal surface of endothelial cells, which was efficiently blocked by using an anti-αv antibody, and that after exposing the cerebral artery to TNF the number of binding events increased. Our results also showed that cRGD peptide alone failed to block MP binding via the integrin αvβ3. This is surprising given the multiple suggested uses of RGD peptide inhibitors^17,24^, but clearly suggests that the RGD peptide-αvβ3 interaction requires polyvalent ligand interactions for optimal adhesion under flow conditions. Interestingly, our data revealed the MP-bound cRGD peptide as a more effective construct to interfere with the adhesive function of αvβ3 integrin than small molecule systems used previously^17,25^.

In this study we raise the possibility that MPs conjugated to specific ligands could also mediate cell binding to the activated endothelium, a process which is important in a number of pathologies, and here could be investigated in an isolated though physiologically relevant manner. For example, previous studies have suggested that the interactions between platelets and endothelial adhesion receptors depends on polyvalent ligand binding^26^, but the molecular basis for this mechanism remains elusive. Early *in vitro* studies by Gawaz *et al*.^6,8^ found that platelet-derived EVs are able to bind to endothelial cells, but the role and mechanisms through which EVs contributed platelet adhesion in their experiments remained obscure^8^. While in this study we did not characterize the cellular source and origin of our endogenous EVs, the majority of EVs in the circulation are of platelet origin^27^. Here, we have demonstrated that a polyvalent ligand interaction between resting platelets and the structurally intact endothelium is mediated by MP-carrying RGD peptides. As such it seems feasible that the interaction between EVs and the endothelium is key for αvβ3 integrin-mediated platelet adhesion within the blood vessel. In pathology, such as ischemic stroke, where increased coagulopathy after infarction is a key event, this mechanism provides the potential for therapeutic intervention.

When EVs from tMCAO rats were delivered into the lumen of the normal cerebral artery we found that platelet adhesion to the endothelium was significantly increased, compared to when we stimulated with EVs obtained from sham rats. Endogenous circulating EVs have been shown to be significantly elevated after ischemic stroke^15,28^, but whether EVs worsen ischemic stroke outcomes via providing a binding nexus at the endothelial surface is yet to be determined. It should be noted that our results with EVs from stroke animals are correlative in nature and we cannot exclude involvement of other mechanisms independent of EV-carried RGD ligands and their interactions, such as complement, which also could be responsible for augmented platelet adhesion. Our western blotting and proteomic analysis revealed the presence of RGD containing integrin ligands, which could be transferred by fusion processes to the surface of endothelial cells to alter adhesion. Likewise circulating EVs also contain miRNA that are able to transduce endothelial cells and alter function^29^. However, the MP-carrying RGD peptides used here show that increased adhesion can be achieved in the absence of EV fusion events.

Collectively, our results demonstrate that MP- and EV-carried RGD proteins and peptides bind in a polyvalent manner with the endothelium to promote platelet adhesion during inflammation. By studying this *ex vivo* we have effectively demonstrated these phenomena under physiologically relevant conditions. Conceptually, this approach could prove of considerable use both manipulating and studying integrin/platelet interaction in key pathological conditions such as stroke, cerebral inflammation and CNS tumors.

## Supplementary information

Supplementary Figures

## Data Availability

We declare that the data supporting the findings of this study are available within the article and its Supplementary Information files and from the corresponding authors upon request.
