# Supplementary material for: Extracellular vesicle integrins act as a nexus for platelet adhesion in cerebral microvessels
Source: Sci Rep. 2019 Nov 1;9:15847. doi: 10.1038/s41598-019-52127-3 (PMC6825169; doi:10.1038/s41598-019-52127-3)
Supplement: Supplementary file 1 — Supplementary Figures [file 41598_2019_52127_MOESM1_ESM.pdf]

SUPPLEMENTARY FIGURES

Extracellular vesicle integrins act as a nexus for platelet adhesion in cerebral microvessels

Zsolt Bagi, Yvonne Couch, Zuzana Broskova, Francisco Perez-Balderas, Tianrong Yeo, Simon Davis, Roman Fischer, Nicola R. Sibson, Benjamin G. Davis, Daniel C. Anthony

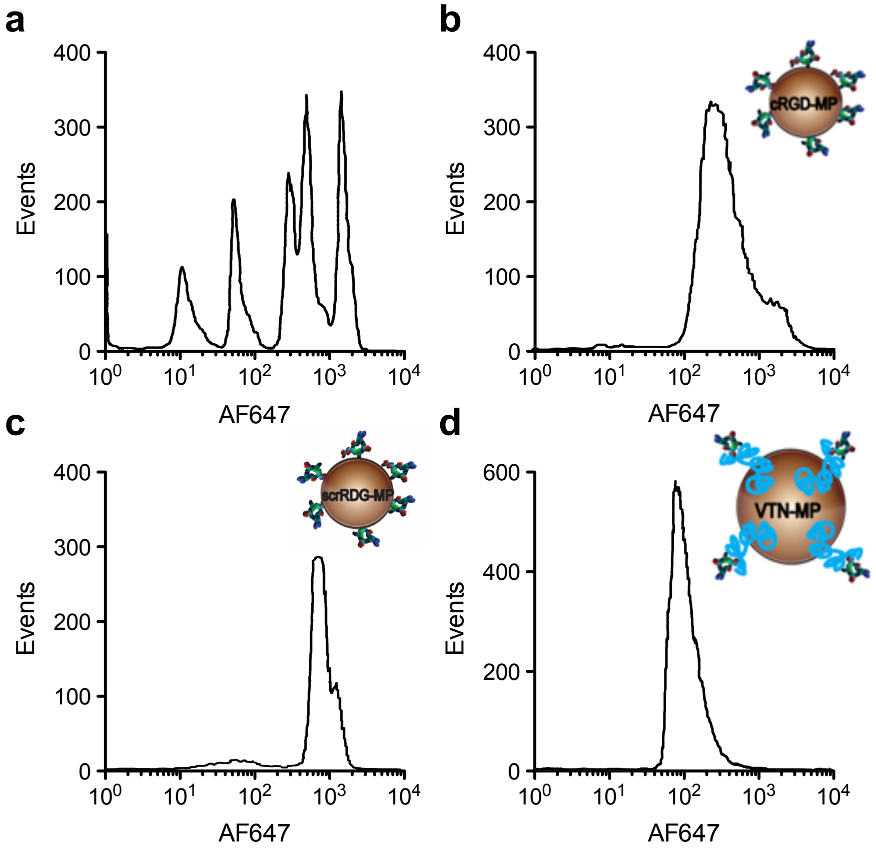

e

Sample	Size (μm)	Surface Density (agent/particle)	Surface Density (agent/μm^2)
cRGD-MP	1.0	146597	46778
scrRGD-MP	1.0	259661	82653
VTN-MP	2.8	17384	706

Supplementary Figure 1. Flow cytometry demonstrating labeling efficiency and size of particles; (a) calibration beads at (SIZES); (b) cRGD-linked MPs; (c) scrambled-RGD-linked MPs; (d) VTN-linked MPs; (e) Qifikit assay demonstrating effective labeling of synthetic MPs (agent/μm^2).

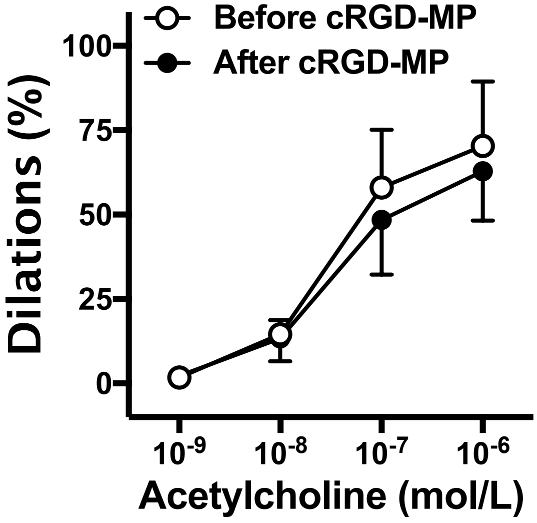

Supplementary Figure 2. Effect of cRGD-MPs on endothelium-dependent vasodilation. Summary data of dilatations in response to cumulative concentrations of acetylcholine (10^{-9} to 10^{-6} M) in isolated and pressurized middle cerebral artery, before and after intraluminal delivery of cRGD-MPs (0.1 µg/ml, N=4).

a

i **ii**

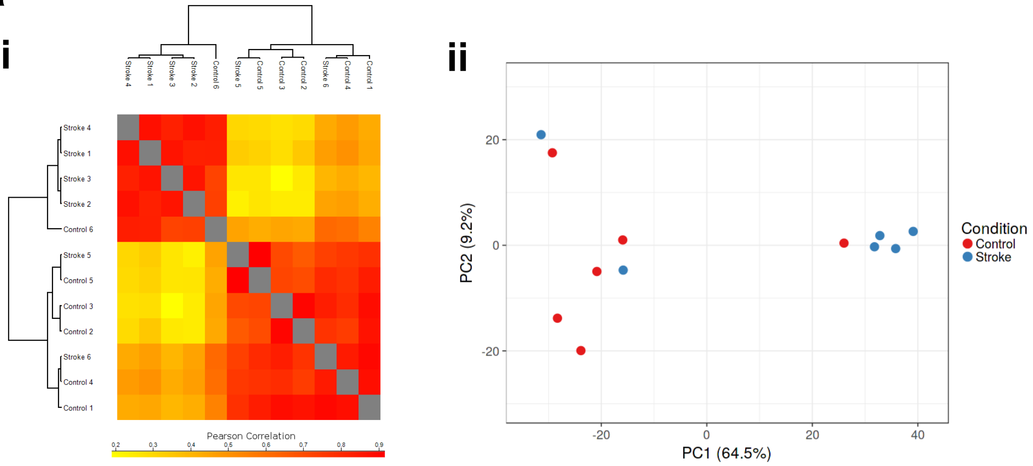

b

i

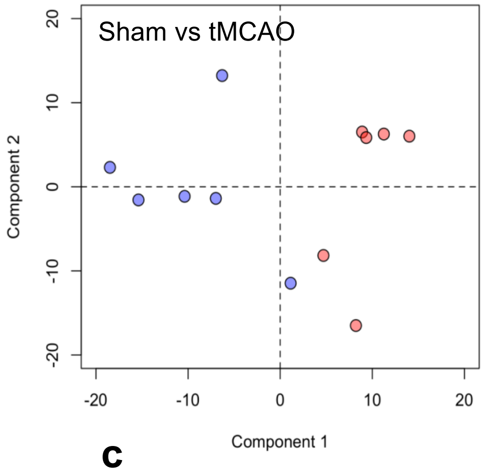

ii

	Acc	SD	Spec	SD	Sens	SD
**model**	0.604167	0.054761	0.790667	0.129509	0.799419	0.130796
**random**	0.466667	0.19245	0.633333	*NA*	0.657285	*NA*

	R2X	SD	R2Y	SD	Q2	SD
**model**	0.78553	0.009868	0.995248	0.004516	0.337541	0.107287
**random**	0.784693	0.017706	0.991262	0.016728	0.241815	0.25721

iii

	Acc	Spec	Sens	R2X	R2Y	Q2
**t-test**	3.46E-10	1.11E-08	1.50E-07	0.680352	0.023228	0.000792
**ks-test**	4.81E-14	6.95E-06	6.68E-07	0.28096	0.000247	9.57E-06

c

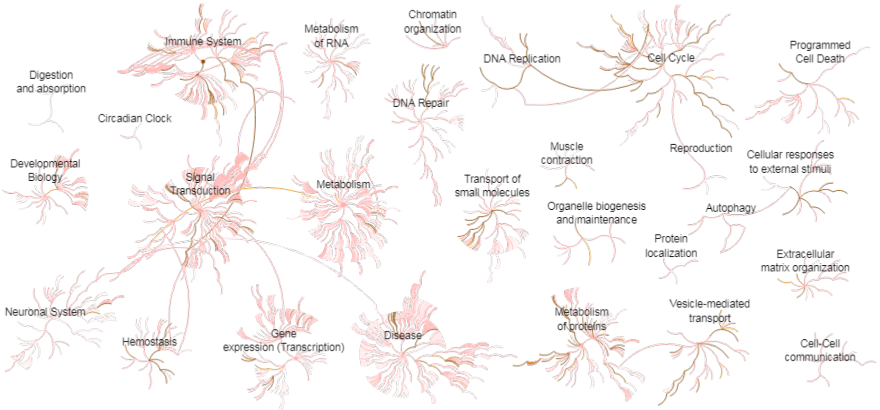

Supplementary Figure 3. Proteomic analysis of EVs in stroke vs. control animals. **a**) Comparison of samples using protein quantitation values determined by liquid-chromatography mass-spectrometry using the 220 proteins that were detected in at least 5 of 6 replicates. (**ai**) Pearson

correlation matrix of samples. Rows and columns are grouped by hierarchical clustering using Euclidean distance. (**aii**) Principal component analysis of samples. The X and Y axis show principal component 1 and 2, which explain 64.5 % and 9.2 % of the total variance respectively. (**bi**) Orthogonal partial-least square determinant analysis (OPLS-DA) scores plot shows good separation between sham and tMCAO animals. (**bii**) The mean accuracy of the OPLS-DA model for sham vs tMCAO is 60.4% while the mean accuracy for the OPLS-DA model for random class assignment is 46.7%. (**biii**) Summary indices of the OPLS-DA model for sham vs tMCAO model. p values indicated are obtained by Kolmogorov-Smirnov test of the sham vs tMCAO OPLS-DA model compared to the random class assignment OPLS-DA model. (**c**) The pathways that are enriched in the EV proteome, which includes vesicle-mediated transport among others.
